# Predictors of condom use and refusal among the population of Free State province in South Africa

**DOI:** 10.1186/1471-2458-12-381

**Published:** 2012-05-28

**Authors:** Thoovakkunon Moorkoth Chandran, Dirk Berkvens, Perpetual Chikobvu, Christiana Nöstlinger, Robert Colebunders, Brian Gerard Williams, Niko Speybroeck

**Affiliations:** 1 Department of Health, Free State, Bloemfontein, 9300, South Africa; 2 Institute of Tropical Medicine, Antwerp, Belgium; 3 Department of Epidemiology and Social Medicine, University of Antwerp, Antwerp, Belgium; 4 South African Centre for Epidemiological Modeling and Analysis, University of Stellenbosch, Stellenbosch, South Africa; 5 Institut de Recherche Santé et Societé (IRSS), Université catholique de Louvain, Brussels, Belgium

## Abstract

**Background:**

This study investigated the extent and predictors of condom use and condom refusal in the Free State province in South Africa.

**Methods:**

Through a household survey conducted in the Free Sate province of South Africa, 5,837 adults were interviewed. Univariate and multivariate survey logistic regressions and classification trees (CT) were used for analysing two response variables ‘ever used condom’ and ‘ever refused condom’.

**Results:**

Eighty-three per cent of the respondents had ever used condoms, of which 38% always used them; 61% used them during the last sexual intercourse and 9% had ever refused to use them. The univariate logistic regression models and CT analysis indicated that a strong predictor of condom use was its perceived need. In the CT analysis, this variable was followed in importance by ‘knowledge of correct use of condom’, condom availability, young age, being single and higher education. ‘Perceived need’ for condoms did not remain significant in the multivariate analysis after controlling for other variables. The strongest predictor of condom refusal, as shown by the CT, was shame associated with condoms followed by the presence of sexual risk behaviour, knowing one’s HIV status, older age and lacking knowledge of condoms (i.e., ability to prevent sexually transmitted diseases and pregnancy, availability, correct and consistent use and existence of female condoms). In the multivariate logistic regression, age was not significant for condom refusal while affordability and perceived need were additional significant variables.

**Conclusions:**

The use of complementary modelling techniques such as CT in addition to logistic regressions adds to a better understanding of condom use and refusal. Further improvement in correct and consistent use of condoms will require targeted interventions. In addition to existing social marketing campaigns, tailored approaches should focus on establishing the perceived need for condom-use and improving skills for correct use. They should also incorporate interventions to reduce the shame associated with condoms and individual counselling of those likely to refuse condoms.

## Background

Globally, the number of people living with HIV and AIDS has been increasing, while the number of new infections and AIDS related deaths has declined slightly [1]. Sub-Saharan Africa, where 67% of the global 33.4 million people living with HIV and AIDS reside, continues to be the worst affected region of the world. In South Africa, more than five million people are living with HIV and AIDS [2]. The overall HIV prevalence in South Africa was estimated at 11.4%, 10.8% and 10.9% of the total population excluding infants under 2 years of age in 2001, 2004 and 2008 respectively [3,4]. There is evidence that condoms are highly effective in reducing sexual transmission of HIV [5]. While they are also the most widely available prevention means they are currently not used to their full potential as a low cost prevention technology.

Recognizing the multifaceted nature of behavioural outcomes [6,7], studies of condom use have considered various levels of influencing factors. Most of the existing, empirically validated AIDS behavioural theories share some overlapping psychological constructs [8]including cognitive factors, beliefs and attitudes towards condoms, as in the Theory of Reasoned Action [9], skills needed to use condoms effectively, as in the Information-Motivation-Behavioural Skills model [10], and social norms as in Social Cognitive Theory [11]. At an intrapersonal level, educational aspirations and students’ performance [12], ability to plan and prepare for condom-use [14], personal coping strategies including alcohol use [14], personality traits such as sensation seeking and impulsivity [15] have been found to be related to condom use. At an interpersonal level relationship variables (partner type) [16], parent–child communication and parental supervision [17] have been shown to be associated with condom use. At an environmental level, workplace or school related peer-pressure and broader contextual factors such as cultural norms or policies also affect condom use. In South Africa, for instance, the interplay between socio-economic factors, service costs, condom availability, condom knowledge and its access and tobacco and alcohol use, were all found to predict demand for condoms [18]. Further, gender disparities in socio-economic status were found to influence women’s ability to negotiate condom use [19,20].

In spite of a recent increase in condom use of about 65%, estimates of HIV incidence in South Africa remain between 1% and 2% (new infections per year) among young people aged 15–20 years [21]. We therefore need a better understanding of factors that hinder and facilitate condom use in order to reduce HIV incidence in the future. The analysis should also explore the reasons why people refuse condom use in spite of prevention campaigns and high levels of knowledge. Studies trying to explain ‘condom refusal’ rather than condom use have been limited to exploring its relation to HIV risk and gender violence [22].

The aim of this study was to investigate the extent and predictors of condom use and condom refusal in the Free State province. We investigate the question as to whether or not the predictors are the same or different for these two outcome variables, by employing and comparing two different and complementary statistical models. Exploring condom refusal in more detail may lead to the development of more nuanced prevention messages and may be used to inform region-specific and nationwide prevention policies.

## Methods

### Study setting

The Free State with nearly 3 million inhabitants is one of the nine provinces in South Africa with a high HIV prevalence, estimated at 12.6% among the population above two years of age in the 2005 and 2008 South African National HIV Prevalence Surveys. As in other provinces, awareness campaigns and condom promotion programs were introduced in 1995 and reported condom use increased from 35% in 2002 to 65% in 2008.

### Sampling method

Data used in this paper were from a cross-sectional study conducted during the first half of 2009 in the Free State province of South Africa, commissioned by the Provincial Department of Health. The Free State is divided geographically into 20 Local Municipalities (LM). A cluster sampling method was used with LM as the primary sampling unit. Each LM was divided into enumeration areas based on the 2001 census [23]. Thirty enumeration areas were selected using a stratified random sample with probability proportional to size. Ten households were selected sequentially starting at a household identified randomly in each sector. In each household only one participant above the age of 17 years was interviewed after his/her written consent was obtained. If there was only one participant in the household, he/she is interviewed. If there were more than one, one respondent was selected randomly. The interviews were conducted by research assistants recruited and trained in interview techniques and in how to fill out the questionnaire. The household interviews were carried out face-to-face in the participant’s language, which was usually the mother tongue of the interviewee. The sample size achieved during the data collection ranged from 273 to 310 per LM and yielded a total sample size of 5,837 participants for the whole province.

### Measurements

To assess predictors of condom use and condom refusal, a questionnaire was developed using Epi-Info version 3.5.1. The development of the questionnaire was guided by a simplified theoretical framework, considering predictors of condoms use shared by relevant behavioural theories such as knowledge, attitudes and beliefs, and socio-cultural norms. The questionnaire containing 110 mostly closed questions was translated from English into Northern Sotho and Afrikaans; the translations were tested in a pilot study and validated. In addition to the two main outcome variables, 19 predictor variables were included in the questionnaire based on the empirical evidence available on factors influencing condom use versus condom refusal. The predictor variables consisted of demographic information (age, gender, ethnic group, marital status, education, employment and type of residence, own and partner’s HIV status), intrapersonal variables (e.g. knowledge, attitudes towards condoms) and contextual variables such as availability, affordability and whether condoms were obtained from public or private sources. Other independent variables were related to health behaviour.

#### Knowledge of HIV and condoms

‘Knowledge of HIV’ was measured by four statements with ‘Agree’, ‘Disagree’ and ‘Don’t know’ responses concerning the curability of HIV, unsafe sex as its cause, treatment of opportunistic infections and benefit of knowing one’s HIV status. Since the assessment of HIV-related knowledge had to remain brief, statements were selected that typically cut across the many areas relevant to HIV-related knowledge. An incorrect or ‘don’t know’ response to any one of these statements was considered as inadequate knowledge.

‘Knowledge about condoms’ was assessed by four statements about the extent to which condoms to prevent sexually transmitted diseases and pregnancy, their free availability in hospitals and clinics, their correct and consistent use and awareness about the existence of female condoms. A wrong response to any of these statements was considered inadequate knowledge.

#### Beliefs and attitudes related to condoms

The belief that condoms can prevent HIV was assessed by the question “Do you believe that the use of condoms can prevent HIV?” The perceived need for condoms was assessed by asking the Yes/No question “Are you in need of a condom to prevent HIV nowadays?”

#### Socio-cultural norms

Stigma or shame related to HIV may also be expressed through socially and culturally grounded attitudes towards condoms. The degree to which participants’ felt that there was shame associated with condom (referred to as condom stigma) was evaluated by five ‘Yes/No’ questions. Participants were asked whether or not they were ashamed of using condoms, to purchase condoms, taking condoms from free distribution points and talking about condoms in general and with their partner. A ‘Yes’-response was considered to exhibit shame associated with condom, which in a social context may be related to ‘condom stigma’.

#### Contextual variables

The questions used to measure contextual variables were: “Are condoms available to you if you need one?” with ‘Yes/No’ response for ‘availability’, “Are condoms affordable to you if you want to buy one?” with ‘Yes/No’ responses for ‘affordability’ and “What is your usual source of condom?” with a ‘Free/Paid source’ response for ‘usual source of condom’.

#### Sexual risk behaviour

Sexual risk behaviour other than not using condoms was assessed using four questions: sexual debut before the age of 15, multiple concurrent sexual partners, frequent change of sexual partners and ever having been forced into sexual intercourse. Any one of these risky behaviours was considered sufficient to categorize a participant as having sexual risk behaviour.

#### Outcome variables

The analyses were conducted with two outcome variables: ‘ever used condom’ and ‘ever refused condom’.

### Analysis

The analysis plan included two complementary statistical models in order to help explore the complexity and interplay between explanatory variables: multivariate logistic regressions and classification trees which is a non-parametric data-mining technique.

Univariate and multivariate logistic regressions were conducted with two dependent variables: ‘ever used condom’ and ‘ever refused condom’ using a survey logistic regression in Stata [24], allowing for the design effects of clustering. In order to capture as much condom use as possible, the variable ‘ever used condom’ was used instead of ‘used condom during last sexual intercourse’ [25].

Classification Trees (CT) were used to explore the influence of the specified predictors on the use or refusal of condoms. CT models are useful tools to explore the relationship between a desired outcome and its determinants [26] and have been used in several disease contexts e.g. malaria [27-29].

The building of a classification tree begins with a root (parent) node, containing the entire set of observations, and then through a process of yes/no questions, generates descendant nodes. Beginning with the first node, containing the complete sample, CT finds the best possible variable to split the node into two child nodes. In order to find a best variable, the software checks all possible splitting variables, as well as all possible values of the variable to be used to split the node, seeking to maximize the average “purity” of the two child nodes. In other words, the child nodes will be as homogeneous as possible with respect to the outcome variables (i.e. condom use and refusal). The splitting is repeated along the child nodes until a terminal node is reached.

In our study, CT may provide additional insights to those obtained from a logistic regression, for a number of reasons. Firstly, as CT works with (non-predefined) interactions in a flexible way it makes it possible to deal with a large number of explanatory variables, as is the case in this study. Standard regression analyses rapidly become unreliable when the dimensionality is very high while CT handles multiple interactions in a more flexible way based on decision trees. In a decision tree analysis, subgroups are obtained by splitting the entire data set by finding the best splitting variables, and they are considered new starting populations, resulting in a natural creation of interactions. For example, the total sample (starting node) may be split in two subgroups according to for instance, income. It is possible that other variables only play a subsidiary role and are thus used as further splitters, in the low income group. This means that an interaction between two variables may be detected in a natural and non-predetermined way as is the case in standard regression techniques.

A second advantage of CT is that it deals with multi-collinearity in an intuitively correct way. Two approximately collinear variables in a logistic regression model can influence their significance levels and may change their association with the outcome variable. In a decision tree the more important of two collinear variables will be selected but the improvement measure attributable to each variable in its role as a either a primary or a surrogate splitter is also computed. Surrogate variables closely mimic and predict the action of primary splitting variables. The values of all these improvements are summed over each node and totalled, and are then scaled relative to the best performing variable. The importance score measures a variable’s ability to perform either as a primary splitter or as a surrogate splitter. If one variable is not selected at several splits because it is the second most important variable each time it may not appear in the tree, but it will appear in the variable importance table, ranking the variables based on their contribution in the construction of the tree. In addition, a tree is comprehensible to a wide audience and results in a clear division of the original sample in groups of high and low risk making it useful to policy makers.

It is still the case that the construction of trees is sometimes unstable. The method of cross-validation can then be used, which consists of dividing the entire sample randomly into N (usually 10) sub-samples, stratified by the response variable. One sub-sample is then used as the test sample and the other N-1 (e.g., nine) are used to construct a large tree. The entire model-building procedure is repeated N times, with a different subset of the data reserved for use as the test dataset each time. Thus, N different models are produced, each one of which can be tested against an independent subset of the data.

The strength of a tree can be indicated by its sensitivity and its specificity. The sensitivity for condom use for example, is calculated to indicate how many of the users are classified as users, meaning that they fall in a terminal node with a proportion of users higher than the average use in the population. The specificity is calculated to indicate how many of the non-users are classified as non-users.

Using different analytical tools (i.e., parametric and non-parametric) can result in interesting insights. For example, in a classical logistic regression, linear combinations are the primary method of expressing the relationships between variables, while in classification trees this relationship does not need to be linear or additive. A classical regression may be more appropriate to quantify linear relationships. A further advantage of a classical regression is the probability level or confidence interval associated with the coefficients in the model. By using the results obtained through CT in a complementary way to those of the parametric models, we combine the strengths of the two methodologies.

### Ethical approval

Ethical approval was obtained from the Research Ethics Committee of the Faculty of Health Sciences, University of Free State, Bloemfontein.(Address: The Chairperson: Ethics Committee, Faculty of health sciences, PO Box 339 (g40), Bloemfontein, South Africa).

## Results

Our sample (n = 5837) consisted of 76% urban and 24% rural residents. There were 57% women and 91% Africans compared to 52% and 87% respectively in the population of the Free State province. Most study participants were young (57% in the age group 18 to 29 years; 27% between 30–39 years, 12% between 40 to 49 years, and 4% were above 50 years). Almost 34% of the participants were married or living together. Most participants were unemployed or students. Seven per cent of the participants had completed higher education (Table 1).

**Table 1:** Sample Distribution of Independent variables

**Predictors variable**	**Count**	**Percent**
**1. Residence Type**		
Formal Urban	3,317	58.7
Informal Urban	973	17.2
Rural	1360	24.1
**2. Gender**		
Male	2,455	42.5
Female	3,318	57.5
**3. Ethnic Group**		
Asian/White	232	4.0
Coloured	291	5.1
African		90.9
**4. Age**		
18 to 29 years	3302	57.3
30 to 39 years	1538	26.7
40 to 49 years	697	12.1
50 and above	229	4.0
**5. Marital status**		
Unmarried	3,466	60.7
Married/Living together	1,931	33.8
Divorced/Widowed	310	5.4
**6. Employment**		
None	2,980	51.5
Student	635	11.0
Temporary employment	834	14.4
Permanent employment	1333	23.1
**7. Education**		
None/Below grade 12	3,354	57.9
Passed Grade 12	2,047	35.3
Degree/diploma	396	6.8
**8. Knowledge about HIV**		
Inadequate	3373	59.3
Adequate	2317	40.7
**9. Knowledge about Condoms**		
Inadequate	3098	54.1
Adequate	2631	45.9
**10. Knowledge about correct use of condoms**		
No	1003	17.3
Yes	4787	82.7
**11. Knowledge about own HIV status**		
No	2635	45.3
Yes	3179	54.7
**12. Knowledge about HIV status of Partner**		
No	3502	60.7
Yes	2271	39.3
**13. Belief that condoms prevent HIV**		
No	634	11.0
Yes	5150	89.0
**14. Perceived need for condoms**		
No	834	14.4
Yes	4951	85.6
**15. Presence of stigma on Condom**		
No	4221	73.2
Yes	1548	26.8
**16. Availability of condoms**		
No	721	12.4
Yes	5086	87.6
**17. Affordability of condoms**		
No	1554	26.8
Yes	4246	73.2
**18. Usual source of condoms**		
Free source	3351	73.6
Paid source	1203	26.4
**19. Sexual risk behaviour**		
No	2905	51.6
Yes	2728	48.4

### Condom use and refusal

About 17% (935 out of 5,563 sample) of the participants never used condoms. Among those who ever used condoms, 38.5% used them always, 29% used them most of the time and 15.5% used them occasionally. Out of 5,764 participants 2,165 (37.6%) refused to use condom in the past. This finding indicates that participants using a condom with some partner had refused its use with other partners.

### Knowledge of HIV, condom and HIV status

Forty one percent had adequate knowledge of HIV, defined as a correct response to all four HIV knowledge related items as shown in Table 1, item 8. Correct responses to the single statements ranged from 70% to 92%. These questions dealt with different aspects of HIV and had a low internal consistency and reliability (Cronbach’s alpha = 0.19). Similarly, the Cronbach’s alpha (=0.33) for the single responses relating to condom knowledge was low. Individual statements concerning knowledge of condoms yielded correct responses ranging from 75% to 98%. Overall adequate knowledge (correct answer to all statements) was 46%, however, most participants (83%) knew how to use condoms correctly. Among the participants, 55% knew their own HIV status and 39% knew the partner’s HIV status (Table 1, items 10–11).

### Beliefs and attitudes related to condoms

The questionnaire revealed that 89% believed that condoms prevent HIV transmission. The perceived need of condoms among the participants was 86% (Table 1, item 18).

### Socio-cultural norms

The ‘Yes’ responses to five different questions, related to ‘shame associated with condoms’ (referred to as condom stigma) ranged between 9% and 13%, but overall 26% responded positively to at least one condom stigma associated question. The Cronbach’s alpha (=0.79) for this measure indicated good internal consistency and reliability of this scale.

### Availability and affordability

As shown in Table 1, the majority of the participants (88%) reported that condoms were available, 73% said they were affordable and 74% procured free condoms from government outlets.

### Sexual risk behaviour

The proportion of participants who reported at least one “sexual risk behaviour” was 48% (Table1). Twenty-three per cent of the participants had their sexual debut before the age of 15 years, 30% reported having more than one partner at the same time, 13% indicated frequent change of partners and 10% reported having been forced into sexual intercourse.

### Predictors of condom use through logistic regression

The univariate analysis revealed that participants who were more likely to use condoms were young compared to elderly, unmarried compared to married, Africans compared to White or Asians, male compared to female, urban compared to rural residents and with grade 12 or higher education compared to below 12 grade. Being a student was also strongly associated with condom use with an odds ratio (OR) = 3.2. Other predictors of condom use were the perceived need for condoms (OR = 14.9), knowledge about correct condom use (OR = 9.1), availability (OR = 5.1), belief in condom as an effective HIV prevention method (OR = 3.0), affordability (OR = 2.9) and purchasing condoms compared to getting them for free (OR = 2.1) (Table 2).

**Table 2:** Univariate and multivariate (adjusted) analysis of variable ‘ever used condom’ using survey logistic regression

**Predictors Variable**	**Unadjusted Odds Ratio (95% CI)**	**Adjusted Odds Ratio (95% CI)**
**1. Residence Type**		
Formal Urban	1	1
Informal Urban	0.95 (0.72-1.27)	1.21 (0.53-2.74)
Rural	0.54 (0.39-0.77)**	0.97 (0.43-2.19)
**2. Gender**		
Male	1	1
Female	0.68 (0.53-0.88)**	0.84 (0.50-1.40)
**3. Ethnic Group**		
Asian/White	1	1
Coloured	1.40 (0.60-3.27)	0.67 (0.10-4.41)
African	2.71 (1.27-5.76)**	0.83 (0.24-2.89)
**4. Age**	0.93 (0.91-0.94)**	0.95 (0.94-0.97)**
**5. Marital status**		
Unmarried	1	1
Married/Living together	0.22 (0.16-0.32)**	0.35 (0.21-0.60)**
Divorced/Widowed	0.29 (0.19-0.44)**	0.64 (0.15-2.71)
**6. Employment**		
None	1	1
Student	3.24 (2.15-4.88)**	2.10 (0.72-6.12)
Temporary employment	1.16 (0.84-1.60)	1.54 (0.69-3.42)
Permanent employment	0.76 (0.57-1.00)*	0.93 (0.51-1.70)
**7. Education**		
None/Below grade 12	1	1
Passed Grade 12	2.75 (2.12-3.54)**	1.10 (0.52-2.31)
Degree/diploma	1.59 (0.98-2.58)*	1.14 (0.33-3.90)
**8. Knowledge about HIV**		
Inadequate	1	1
Adequate	1.93 (1.49-2.50)**	1.58 (0.87-2.86)
**9. Knowledge about Condoms**		
Inadequate	1	1
Adequate	1.53 (1.17-2.02)**	1.42 (0.66-3.07)
**10. Knowledge about correct use of condoms**		
No	1	1
Yes	9.06 (6.74-12.17)**	2.50 (1.17-5.35)*
**11. Knowledge about own HIV status**		
No	1	1
Yes	1.75 (1.33-2.30)**	1.73 (0.94-3.20)*
**12. Knowledge about HIV status of Partner**		
No	1	1
Yes	1.50 (1.20-1.89)**	1.00 (0.64-1.58)
**13. Belief that condoms prevent HIV**		
No	1	1
Yes	3.00 (2.33-3.87)**	0.97 (0.39-2.44)
**14. Perceived need for condoms**		
No	1	1
Yes	14.90 (9.73-22.79)**	1.65 (0.81-3.37)
**15. Presence of stigma on Condom**		
No	1	1
Yes	0.33 (0.24-0.45)**	0.53 (0.30-0.94)*
**16. Availability of condoms**		
No	1	1
Yes	5.12 (2.76-9.49)**	2.41 (1.32-4.41)**
**17. Affordability of condoms**		
No	1	1
Yes	2.88 (2.15-3.84)**	1.13 (0.67-1.92)
**18. Usual source of condoms**		
Free source	1	1
Paid source	2.13 (1.07-4.24)*	1.08 (0.46-2.55)
**19. Sexual risk behaviour**		
No	1	1
Yes	1.35 (1.05-1.74)**	1.70 (1.10-2.61)*

***p < .01, **p < .05, *p < .10.

The multivariate logistic regression analysis reported in Table 2, showed that the young (compared to elderly) and married (compared to unmarried) were more likely to use condoms. Other significant predictors included knowledge of own HIV status, sexual risk behaviour, as well as knowledge about the correct use of condoms and condom-availability.

### Predictors of condom refusal through logistic regression

The univariate analysis revealed that individuals with condom stigma compared to those without (OR = 2.56), elderly compared to young participants, participants who were divorced or widowed (OR = 1.96) compared to unmarried and those who had low education compared to high education were more likely to refuse condoms. Belief in condoms as effective HIV prevention method showed a significant negative relation with condom refusal (Table 3). The adjusted multivariate model indicated a positive relation with condom stigma and condom affordability. The variable ‘presence of risky behaviour’ correlated positively with both condom use and condom refusal. Perceived need of condoms, and knowledge about condoms, and their correct use showed a significant negative relation with condom refusal (Table 3).

**Table 3:** Univariate and multivariate (adjusted) analysis of the variable ‘ever refused condom’ using survey logistic regression

**Predictor variable**	**Unadjusted Odds Ratio (95% CI)**	**Adjusted Odds Ratio (95% CI)**
**1. Residence Type**		
Formal Urban	1	1
Informal Urban	0.75 (0.50-1.12)	0.77 (0.48-1.25)
Rural	0.90 (0.57-1.42)	0.83 (0.46-1.49)
**2. Gender**		
Male	1	1
Female	0.94 (0.75-1.16)	0.97 (0.69-1.36)
**3. Age**	1.02 (1.01-1.04)*	1.02 (1.00-1.03)
**4. Marital status**		
Unmarried	1	1
Married/Living together	1.05 (0.81-1.37)	0.94 (0.67-1.32)
Divorced/Widowed	1.96 (1.41-2.27)**	1.50 (0.95-2.37)
**5. Employment**		
None	1	1
Student	0.88 (0.67-1.15)	1.05 (0.70-1.57)
Temporary employment	0.89 (0.68-1.18)	0.82 (0.59-1.13)
Permanent employment	0.73 (0.58-0.93)	0.67 (0.48-0.94)*
**6. Education**		
None/Below grade 12	1	1
Passed Grade 12	0.90 (0.64-2.28)	1.25 (0.78-2.01)
Degree/diploma	0.45 (0.27-0.76)*	0.50 (0.25-1.00)
**7. Knowledge about HIV**		
Inadequate	1	1
Adequate	0.60 (0.46-0.79)**	0.83 (0.63-1.10)
**8. Knowledge about Condoms**		
Inadequate	1	1
Adequate	0.45 (0.32-0.63)**	0.58 (0.38-0.87)*
**9. Knowledge about correct use of condom**		
No	1	1
Yes	0.35 (0.25-0.48)**	0.63 (0.46-0.88)*
**10. Knowledge about own HIV status**		
No	1	1
Yes	0.74 (0.61-0.91)*	0.85 (0.66-1.08)
**11. Knowledge about HIV status of Partner**		
No	1	1
Yes	0.77 (0.63-0.94)*	1.09 (0.84-1.42)
**12. Belief that condoms can prevent HIV transmission**		
No	1	1
Yes	0.34 (0.21-0.53)**	0.64 (0.38-1.08)
**13. Perceived need for condoms**		
No	1	1
Yes	0.25 (0.18-0.36)**	0.34 (0.23-0.49)**
**14. Condom stigma**		
No	1	1
Yes	2.56 (1.88-3.48)**	1.89 (1.37-2.59)**
**15. Availability of condoms**		
No	1	1
Yes	0.59 (0.36-0.95)*	0.91 (0.55-1.52)
**16. Affordability of condoms**		
No	1	1
Yes	0.84 (0.60-1.18)	1.54 (1.18-2.02)**
**17. Sexual risk behaviour**		
No	1	1
Yes	1.14 (0.93-1.39)	1.32 (1.08-1.62)*

***p < .01, **p < .05, *p < .10.

### Predictors of condom use through classification trees

**Figure 1:**
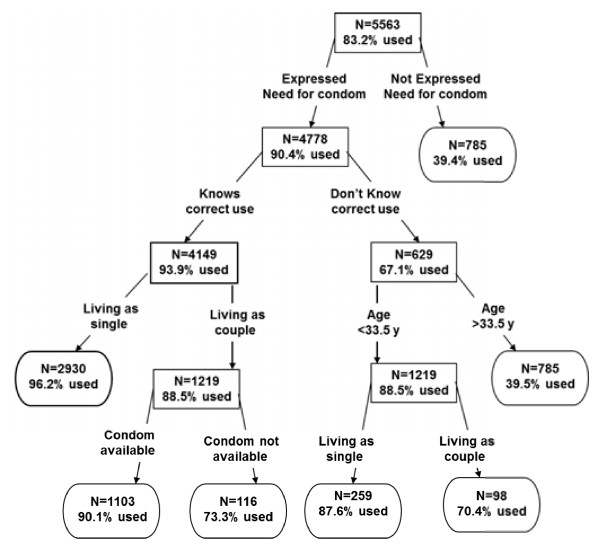
**Classification Tree with ‘ever used condom’ as a response variable.** Note: Each node is characterised by the number of individuals (N) in a subgroup and the proportion of these N individuals using condoms (within the subgroup).

Figure 1 shows the classification tree for the response variable ‘ever used condoms’. The perceived need of condoms was the first predictor of condom use according to the overall discriminatory power of the CT analysis (Table 4), followed by knowledge of its correct use, availability, younger age and living as single.

**Table 4:** Relative importance (relative to the most important, getting an importance of 100) of explanatory variables with ‘ever refused condoms’ and ‘ever used condoms’ as response variables (order according to the importance of ‘ever use condoms’)

**Variable**	**Ever used condom**	**Ever refused condom**
Perceived need for condoms	100	0
Knowledge about correct use of condoms	43.6	0
Availability	39.36	0
Age	37.25	5.25
Marital status	17.58	0
Employment	4.01	0.24
Affordability	1.49	0
Education	1.25	1.05
Knowledge about HIV status of partner	0.12	4.35
Presence of stigma on condom	0	100
Presence of sexual risk behaviour	0	37.52
Knowledge about own HIV status	0	12.01
Knowledge about condoms	0	9.49
Knowledge about HIV	0	3.81
Gender	0	1.9
Ethnic group	0	0
Source of condoms	0	0
Belief that condoms can prevent HIV	0	0
Residence type	0	0

Note: Importance, for a particular variable, is the sum, across all nodes in the tree, of the improvement scores between this variable and the best splitter at a particular node. This means that a variable that does not occur in the tree because always “second best” and not selected as the main splitter, can occur as very important.

Note that importance of a particular variable expresses how important it is relative to the most important variable (getting a score of 100). Figure 1 shows that the study population in which 83% ‘ever used’ condoms, was first partitioned between those with and without a perceived need for condoms (86% versus 14%). Condom use among those with perceived need was 90% compared to 39% among those without. The sub-group with perceived need was further partitioned using the variable ‘knowledge of correct use’ and condom use was 94% for people with knowledge and 67% for people without knowledge. The sub-group ‘with correct knowledge’ was split based on marital status, and living single (96% using condoms) emerged as an important predictor.

Among study participants living in a couple, 88% reported that they used condoms. The perceived availability of condoms was related to condom use (90% if condoms were available and 73% if not). Younger age predicted condom use for the subclass ‘without correct knowledge’ (88% against 39%). Marital status or living single (88% against 70%) was significant in the sub-group of younger age (Figure 1).

The sensitivity and the specificity of the “condom use” tree were 94% and 57% respectively.

### Predictors of condom refusal through classification trees

**Figure 2:**
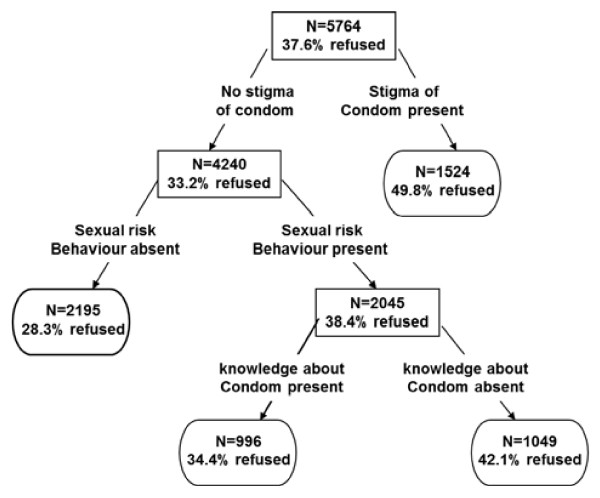
**Classification Tree with ‘refusing condom’ as a response variable.** Note: Each node is characterised by the number of individuals (N) in a subgroup and the proportion of these N individuals refusing condoms (within the subgroup).

The CT analysis showed that condom stigma was the strongest predictor of those that said that they had ever refused to use a condom (Figure 2 and Table 2). Sexual risk behaviour, knowledge of one’s HIV status and that of the partner, knowledge about condoms and older age were less important (Table 2). The classification tree (Figure 2) showed that 38% of the study population had ever refused condoms and the subgroup that reported condom stigma (50% used condoms in this group) was more likely to refuse condoms compared to people without stigma (32%). Among the sub-group that had not experienced condom stigma in relation to condoms, sexual risk behaviour emerged as an important determinant of condom refusal. The classification tree further revealed that low knowledge of condoms increased the likelihood of refusing condoms (42% with adequate knowledge versus 34% with low knowledge refused condoms) (Figure 2).

The sensitivity and the specificity of the “condom refusal” tree were 47% and 70% respectively.

## Discussion

This study analysed the extent and the determinants of both condom use and condom refusal. The overall rates of condom use we found in this study among the Free State population - i.e., 61.3% used condom during last sexual intercourse - compare well with the findings (64.8%) of the 2008 National HIV Prevalence Survey. The geographic and socio-economic features of people who were more likely than others to use condoms were similar to those reported in other studies [30].

The two different analytical techniques, logistic regressions and classification trees, used in this paper, make different assumptions about data and have different strengths. The logistic regression models are useful in determining factors that are associated with the response variable, in the whole population. On the other hand, the classification trees divide the sample in two according to a cut-off value and then further analyses these two sub-populations. Splitting a sample in two results in two specific subsamples and in each of the segments or sub-samples, determinants can play a different role than in the general, initial population. The results of the two logistic regressions and CT are therefore not always the same.

Results of both methods indicate that different underlying constructs may partially influence condom use and condom refusal. As shown, only the variables knowledge about correct condoms use and sexual risk behaviour were associated with the respective outcome variable (condom use versus condom refusal) in the multivariate model, whereas the CT suggested that perceived need for a condom and knowledge of correct condom use were most influential for condom use, compared to condom stigma and sexual risk behaviour for condom refusal. This indicates that contextual factors such as societal norms should be considered more important in explaining condom refusal, whereas individual factors account for actual condom use. This has implications for developing prevention messages.

The regression results indicate that this research like much research in Africa (and elsewhere) finds that condom use and refusal responds to many determinants at multiple levels. The non-parametric classification tree allows further exploring the complexity related to condom use and refusal. To our knowledge this is the first time that such a model is adopted for studying condom use.

The multivariate models reveals factors that predict the behavioural outcome in the whole population, while the CT help detecting segments in the population that have specific prevention needs. Segmenting populations supports decision makers in targeting their efforts to specific subgroups.

While we do not claim that one technique is superior over the other, the strength of this study lies in reporting results using two methodologies, thereby increasing the study’s rigour and achieving a more comprehensive assessment of condom use and condom refusal determinants.

The CT results for example show that in the specific subgroup that knows about condom use and lives as a couple, condoms are not available, condom use is 73% compared to a 90% use when condoms are available. Furthermore, when individuals do not know how to correctly use condoms this leads to lower use (39%) in especially the group older than 33.5 years of age and less in the younger group (88%).

The CT model also provides the relative importance of the variables. The following five variables had the highest discriminatory power in relation to condom use: perceived need for condom, knowledge about correct use of condom, availability, age and marital status. All these variables were also significant in the multivariate logistic regression, with the exception of “perceived need” for condoms, which was significant only in the univariate analysis.

The CT, at the first split, revealed indeed a strong difference in condom use between those with and without a perceived need for condoms. Of the respondents with a perceived need, 39% used condoms, while of the respondents without a perceived need, 90% used condoms. The univariate logistic regression models also indicated that a strong predictor of condom use was its perceived need. However, when controlling for other variables in the multivariate analysis this variable did not remain significant. This may indicate one of the characteristics of the CT, i.e., if a variable on its own explains a high degree of the variability, it will be used as a first split and appear as a strong univariate predictor. The multivariate analysis allows for assessing the effect, while controlling for other variables.

Using the CT results to assess the relative importance of a predictor, indicated that knowledge of correct condom use was shown to be the second strongest predictor. This knowledge may result in additional condom user confidence.

Our findings show that especially older people, and those who are married or living together do not use condoms, which concur with earlier research finding that couples with stable relations are less likely to use condoms [31]. HIV transmission risk in such relations is dependent on knowing the disease status of both partners and strictly adhering to the ‘be faithful’ prevention strategy, which may be subject to false assumptions. HIV- and condom knowledge and belief in the ability of condoms to prevent HIV were non-significant in predicting condom use in the multivariate models and non-important in the CT. This corroborates the contention that knowledge, belief and attitude as such may not be sufficient to achieve behaviour change, calling for multi-level models integrating more comprehensive perspectives. Such a multilevel approach has been used in Kenya and Zambia for instance [32,33]. In Zambia, evidence showed that in addition to individual factors community-level factors can be important and that condom-promotion efforts should pay attention to community-level social norms, population trends, informal social relationships and interpersonal communication. Findings of the study in Kenya also support the relation seen in this study between condom use and age and marital status.

The CT also allows segmenting the population in terms of the condom refusal. It indicates that refusal is especially high when stigma is present; half of this group refusing the use of a condom, while one in three refused the use when stigma was not present. When this stigma is not present, refusal is higher in those with sexual risk behaviour and especially when knowledge of condoms is absent (42% with adequate knowledge versus 34% with low knowledge refused condoms). Such segmentations through interactions are a natural outcome of CT and complement the aforementioned multivariate models. It suggests that knowledge about condoms and sexual risk behaviour are important, but mainly in the group that does not report shame associated to condoms.

In the CT analysis the following top five variables were related to condom refusal: shame associated with condoms, sexual risk behaviour, knowledge about own HIV status, knowledge about condoms, and older age. Shame associated with condoms, sexual risk behaviour and knowledge about condoms as influencing factors were corroborated by the multivariate parametric logistic regression. Affordability of condoms did not turn out to be significantly related to condom refusal in the univariate model and the CT model. However, this variable is significant with an odds ratio greater than one in the multivariate analysis. This finding can be explained by a strong relation between affordability and availability. Controlling for variables such as availability in particular but also stigma and knowledge of correct use makes affordability significant. The significant effect of affordability may indicate that where there is considerable ambivalence about condoms (affordability but also availability) there will be more opportunities for refusal when condoms are affordable than when they are not affordable.

The importance of condom stigma for condom refusal may be explained by its association with HIV stigma. A body of literature shows that HIV-related stigma acts as a strong barrier to actual condom use [34]. This clearly demonstrates the influence of cultural values and social norms in adopting safer sex behaviours [35].

Understanding the reasons behind the refusal to use condoms is particularly important in South Africa because further improvement from its current level of use require innovative and targeted interventions.

The strongest predictor of condom refusal observed in this study, i.e. shame associated with condoms in interaction with other variables stresses the need for changing socio-cultural norms. The strong association of condom refusal with sexual risk behaviour, especially in the group where shame was not expressed, reporting multiple partners, and frequent partner change may require effective counselling.

The social norms and cultural values expressed as shame associated with condom use that may link using condoms to taboo behaviours such as promiscuous sex may lead to condom refusal even in the presence of other factors facilitating condom use (e.g., knowledge of HIV and condom, its availability and affordability and belief that condom can prevent HIV). Additionally, the in-depth exploration of ‘condom refusal’ identified sexual relationships where condom use may be perceived as less important because partners know their HIV status and live in stable relationships. Since heterosexual HIV transmission for both men and women often takes place within marriage or cohabitation, carefully tailored messages would also be needed here [36].

The tree sensitivity and specificity for condom refusal are lower than the tree sensitivity and specificity for condom use, indicating that the variables used assist better in detecting condom users than condom refusers.

This study is subject to some limitations: Data on sexual behaviour were self-reported, thus a social desirability bias may apply, as is generally the case in studies using self-reported data to assess sexual risk behaviour. Another limitation is the issue of causality, as the study uses life time outcome measures with predictors measured at the time of study. Moreover, we did not ask how frequently respondents changed partners and this could have provided further useful insights.

## Conclusions

The importance of the perceived need for condoms found in this study stresses the need for tailored approaches delivered in addition to broadly based social marketing campaigns for condoms [37]. The success of condom promotion programs in South Africa through agencies such as Khomonani, Soul City, Soul Buddyz and Love life is at least partly attributable to social marketing. In South-Africa, awareness campaigns so far have increased the use of condoms among most at risk populations, but other groups also could benefit. Based on our findings, i.e. related to shame associated with condom use and increasing perceived need for condoms we provide specific recommendations for targeted condom promotion.

As identified through the CT those respondents already practicing safer sex and currently not perceiving the need for condoms (mostly older couples) should be motivated to know their status and stay safe. There is also a need for promotion of social acceptance of condoms among married couples, which has been absent from most campaigns so far [38]. Young people already using condoms should be encouraged to maintain their behaviour by providing appropriate knowledge and strengthening their skills for using condoms correctly and consistently. People with sexual risk behaviour are distributed across all age groups and they may be in need of both targeted social marketing and personal counselling to overcome individual barriers to condom-use. For the latter, service providers play a crucial role in carefully messaging this. They are key to promoting condoms as part of a standard package of prevention measures, which contributes to de-stigmatizing and normalizing their use [39].

While findings in our study refer mainly to individual level factors, it is clear that in order to change social and cultural norms in relation to condom stigma, comprehensive social interventions based on social-ecological models are useful [40]. The subgroup that refuses condoms, mainly due to shame associated with condoms could benefit from such interventions. Reduction of HIV-related stigma could be expected to delink condoms from taboo sexual behaviour, resulting in people being less ashamed when talking about or using condoms. In line with WHO and other agencies, the South Africa’s National Strategic Plan 2007–2011, supports a combination of approaches to prevent HIV transmission and most important actions are condom promotion and HIV counselling and testing [41]. Recommendations of our study can be incorporated into the one-to-one pre-test counselling sessions, helping the Free State Health Department to improve the social marketing of condoms and develop targeted interventions to combat stigma.

## Competing interests

The authors declare that they have no competing interests.

## Authors’ contributions

TMC prepared the research protocol, procured funding from Free State Department of Health for the study, conducted the household survey, prepared data for the analysis, and participated in writing and reviewing the manuscript. PC participated in conducting the research, analysed the data and participated in the report writing. NS was the supervising investigator, conducted the data analyses, wrote the first draft and was the lead in the reviewing process. DB, RC, CN, and BGW substantially contributed to the interpretation of data, drafting the article critically for important intellectual content, and approved of the final version. All authors read and approved the final manuscript.

## Pre-publication history

The pre-publication history for this paper can be accessed here:

http://www.biomedcentral.com/1471-2458/12/381/prepub
